# The role of depression in the association between mobilisation timing and live discharge after hip fracture surgery: Secondary analysis of the UK National Hip Fracture Database

**DOI:** 10.1371/journal.pone.0298804

**Published:** 2024-04-04

**Authors:** R. Milton-Cole, A. Goubar, S. Ayis, M. D. L. O’Connell, M. T. Kristensen, F. B. Schuch, K. J. Sheehan

**Affiliations:** 1 Department of Population Health Sciences, School of Population Health and Environmental Sciences, King’s College London, London, United Kingdom; 2 Department of Physical- and Occupational Therapy, Copenhagen University Hospital, Bispebjerg-Frederiksberg and Department of Clinical Medicine, University of Copenhagen, Copenhagen, Denmark; 3 Department of Sports Methods and Techniques, Federal University of Santa Maria, Santa Maria, Brazil; 4 Faculty of Health Sciences, Universidad Autónoma de Chile, Providencia, Chile; 5 Institute of Psychiatry, Federal University of Rio de Janeiro, Rio de Janeiro, Brazil; 6 Bone and Joint Health, Blizard Institute, Queen Mary University of London, London, United Kingdom; Policlinico Universitario A. Gemelli IRCCS - Universita Cattolica del Sacro Cuore Roma, ITALY

## Abstract

**Purpose:**

The aim was to compare the probability of discharge after hip fracture surgery conditional on being alive and in hospital between patients mobilised within and beyond 36-hours of surgery across groups defined by depression.

**Methods:**

Data were taken from the National Hip Fracture Database and included patients 60 years of age or older who underwent hip fracture surgery in England and Wales between 2014 and 2016. The conditional probability of postsurgical live discharge was estimated for patients mobilised early and for patients mobilised late across groups with and without depression. The association between mobilisation timing and the conditional probability of live discharge were also estimated separately through adjusted generalized linear models.

**Results:**

Data were analysed for 116,274 patients. A diagnosis of depression was present in 8.31% patients. In those with depression, 7,412 (76.7%) patients mobilised early. In those without depression, 84,085 (78.9%) patients mobilised early. By day 30 after surgery, the adjusted odds ratio of discharge among those who mobilised early compared to late was 1.79 (95% CI: 1.56–2.05, p<0.001) and 1.92 (95% CI: 1.84–2.00, p<0.001) for those with and without depression, respectively.

**Conclusion:**

A similar proportion of patients with depression mobilised early after hip fracture surgery when compared to those without a diagnosis of depression. The association between mobilisation timing and time to live discharge was observed for patients with and without depression.

## Background

The average length of hospital stay for hip fracture surgery in the United Kingdom (UK) is 21.4 days (Standard Deviation (SD) 19.9) with a range of 12 to 41.9 days [1, 2]. Patients in England with hip fracture had the longest length of hospital stay when compared to ten other high-income countries around the world [2]. Delayed discharge can have negative consequences including complications, decline in mood, social isolation, decline in functional ability, admission to nursing/residential care, or death [3]. This was reflected in a qualitative study in which patients admitted to hospital for hip fracture surgery reported going home as their main goal [4]. The Chartered Society of Physiotherapy’s standards of rehabilitation for hip fracture patients recommends all patients are mobilised on the day of, or the day after, their surgery [5]. This is because early mobilisation (mobilisation within 36 hours of surgery) is associated with increased rates of hospital discharge within 30 days of hip fracture surgery, among other positive outcomes [6]. What’s more, previous research shows early mobilisation results in a two-fold increase in the odds of being discharged home, compared to late mobilisation, by 30-days postoperatively when adjusting for the competing risk of death [6]. The magnitude of this association varies across subgroups of patients who also present with delirium, dementia, and who are admitted from residential care [6, 7].

Following hip fracture, depression contributes to worse functional outcomes, higher chance of being placed in a care home, and higher mortality rates compared to those without depression [8, 9]. Depression has also been associated with longer hospital stays after hip fracture surgery [10, 11]. These poor outcomes may relate to a negative association between depression and motivation to participate in physical rehabilitation [12]. However, it is not known whether the association between early mobilisation and time to discharge varied by a depression diagnosis. Should the association vary, there may be a need to screen for depression and/or consider approaches to improve the rate of early mobilisation among older adults with depression after hip fracture surgery. Therefore, the aim of this study was to compare the conditional probability of discharge, a conservative measure of time to event analyses, after hip fracture surgery between patients mobilised within and beyond 36-hours of surgery in England and Wales across groups defined by depression.

## Methods

### Dataset

Data from the National Hip Fracture Database (NHFD) was linked to the Hospital Episode Statistics for England and the Patient Episode Database for Wales. The dataset, linkage, cleaning, and validation have previously been described [6]. Briefly, the NHFD is upheld by the Royal College of Physicians and collects information on patients’ characteristics and care received for all patients who were hospitalized with a hip fracture in England and Wales [13]. Additional databases supplied data on comorbidities, ethnicity, deprivation, and mortality [6]. For the technical report specifying the data linkage process including record and patient level exclusions, see S1 File included in Sheehan, Goubar [6].

### Patient population

Patients 60 years of age or older who underwent surgery for a nonpathological, first hip fracture in England and Wales between 1^st^ January 2014 and 31^st^ December 2016; whose data were entered into the National Hip Fracture Database (NHFD).

### Variables

#### Depression

Depression was determined by the presence of an International Classification of Diseases (ICD) code for depression in the patients record during the hip fracture admission, or during a hospitalisation in the year prior to hip fracture admission (ICD-10 F204, F32, F33, F34, F43) [14]. It is likely depression pre-dated the hip fracture event, however, for a small minority of patients they might have been diagnosed during the hip fracture admission and therefore, after their hip fracture event.

#### Exposure

The exposure variable was mobilisation timing categorised as early and late mobilisation. Early mobilisation was defined as mobilisation occurring on the day of or day after (within 36 hours) a patient’s hip fracture surgery and late mobilisation was mobilisation occurring two days or more after (beyond 36 hours) a patient’s hip fracture surgery. Mobilisation was defined by the NHFD, with or without assistance, as the observed ability to sit or stand out of bed (6).

#### Outcome

The outcome was time to discharge from hospital after hip fracture surgery. The competing event was death prior to discharge. Censored events included transfers to another acute hospital/unit or loss to follow up (after 30 days).

#### Confounders

The variables in the adjustment set included in the regression models were age [15]; sex (female, male) [16]; ethnicity (White, Black or mixed Black, Asian or mixed Asian, Other mixed background) [17]; deprivation (Index of Multiple Deprivation decile groups) [18]; number of comorbidities (which may be associated with the exposure–mobilisation timing (delirium, dementia, hypotension) or as proxies for medical instability/contraindications (cardiopulmonary conditions)) [19]; American Society of Anaesthesiologists (ASA) grade (0–4) [20]; prefracture residence (Own home/sheltered housing, Nursing care/residential care) [15]; fracture type (Intracapsular, Intertrochanteric, Subtrochanteric) [15]; mobility prior to hip fracture (indoor only, indoor and outdoor, none) [21]; hospital surgical volume (low (quartile of fewest cases), medium (second and third quartile), or high (fourth quartile) [22]; timing of surgery (within 36-hours, not within 36-hours) [23]; day of admission (weekday, weekend) [22]; calendar year of admission (2014, 2015, 2016) [22].

### Statistical analysis

Patient characteristics are presented by the presence and absence of a diagnosis of depression and mobilisation timing. Chi-square test and Mann-Whitney U test were used to compare distributions across exposure groups for those with and without a diagnosis of depression.

We estimated separately the conditional probability function (CPF) of postsurgical live discharge as functions of inpatient days after surgery for patients mobilised early and patients mobilised late, across groups defined by depression [24]. The conditional probability estimates the probability of discharge at 30-days postoperatively conditional on patients being alive and in hospital, by considering discharges in the numerator and both deaths and discharges in the denominator [25]. We compared the estimated CPFs of patients mobilised within 36-hour of surgery and those mobilised beyond this time period, within each depression subgroup using the Pepe-Mori 2-sample test [26].

We estimated the odds ratios (OR) for the association between mobilisation timing and the CPF of live discharge at day 3, 4, 6, 8, 12, 16, 20, 24, 30. The ORs demonstrate whether the CPF of live discharge at 30 days postoperatively differed between those mobilised early compared to those mobilised late. We used pseudo-values from a jackknife of the CPF in a generalized estimating equation (equivalent method to a proportional odds or ordered logistics regression approach) including depression by mobilisation as an interaction term, and accounting for the adjustment set described above [27]. The depression by mobilisation timing interaction was assessed by the Wald chi-squared test. The models were assessed for regression model assumption violations and the covariate correlations in the models. Analyses were conducted in Stata 16 and R (version 4.2.3) [28, 29].

### Sensitivity analyses

The sensitivity of complete case analyses to the influence of missing data in exposure, subgroup, and confounding variables were assessed in a series of analyses using multiple imputation by chained equations [30].

## Results

### Patient characteristics

Data were analysed for 116,274 patients with complete data for both exposure and outcome variables. A diagnosis of depression was present in 9,659 (8.3%) patients. Of those patients 7,494 (77.6%) were female, median age was 82 (IQR: 75–88), 2,219 (23.5%) had an ASA grade I or II, and 6,487 (68%) were mobile indoors and outdoors prefracture (Table 1). In those with depression, 7,412 (76.7%) patients mobilised early (p<0.001 (compared to those with depression who mobilised late)). By day 30 after surgery, 5,019 (52%) stays ended with live discharge, 345 (3.6%) stays ended with hospital death, 3,324 (34.4%) were lost to follow up as they were discharged to another acute hospital or a rehabilitation unit and 1,197 (12.4%) had not been discharged from hospital by the end of follow up at 30 days (Table 2).

**Table 1:** Characteristics of 116,274 patients surgically treated for non-pathological first hip fracture overall and by timing of mobilisation and depression diagnosis.

Variables	Diagnosis of Depression (n = 9,659)		No diagnosis of depression (n = 106,615)	
	Mobilised early ^a^ (n = 7,412)	Mobilised late ^a^ (n = 2,247)	Mobilised early ^a^ (n = 84,085)	Mobilised late ^a^ (n = 22,530)
**Age at admission (years), median (IQR)**	82.0 (74.0–88.0)	83.0 (76.0–88.0) *	84.0 (77.0–89.0)	85.0 (79.0–90.0) *
**Number of comorbidities, median (IQR)**	2.0 (1.0–3.0)	2.0 (1.0–3.0) *	1.0 (1.0–2.0)	2.0 (1.0–3.0) *
**Sex**				
Women	5,755 (77.7)	1,735 (77.2)	60,959 (72.5)	15,870 (70.5)
Men	1,647 (22.3)	513 (22.8)	23,127 (27.5)	6,625 (29.5) *
**Ethnicity** ^**b**^				
White	6,258 (99.1)	1,812 (99.0)	69,616 (98.6)	17,743 (98.1)
Non-white	60 (0.9)	18 (1.0)	1,021 (1.4)	345 (1.9) *
**Deprivation**				
least deprived 10%	729 (9.9)	239 (10.7)	7,095 (8.5)	1,985 (8.9)
less deprived 10–20%	694 (9.4)	233 (10.4)	6,913 (8.3)	2,018 (9.0)
less deprived 20–30%	737 (10.0)	241 (10.8)	7,574 (9.1)	2,194 (9.8)
less deprived 30–40%	730 (9.9)	236 (10.6)	8,225 (9.9)	2,331 (10.4)
less deprived 40–50%	805 (10.9)	196 (8.8)	8,705 (10.4)	2,404 (10.8)
more deprived 40–50%	753 (10.2)	255 (11.4)	9,286 (11.1)	2,475 (11.1)
more deprived 30–40%	810 (11.0)	232 (10.4)	9,110 (10.9)	2,410 (10.8)
more deprived 20–30%	760 (10.3)	231 (10.3)	8,951 (10.7)	2,248 (10.1)
more deprived 10–20%	701 (9.5)	193 (8.6)	8,994 (10.8)	2,203 (9.9)
most deprived 10%	648 (8.8)	176 (7.9) †	8,579 (10.3)	2,049 (9.2) *
**ASA Grade** ^**c**^				
0–1	1,897 (26.2)	321 (14.7)	26,908 (32.8)	4,274 (19.4)
2	4,378 (60.5)	1,394 (63.9)	45,826 (55.9)	13,130 (59.7)
3–4	958 (13.2)	467 (21.4) *	9,292 (11.3)	4,594 (20.9) *
**Prefracture Residence**				
Own home/sheltered housing	5,353 (74.4)	1,327 (61.5)	69,401 (84.0)	16,086 (73.7)
Nursing care/residential care	1,838 (25.6)	829 (38.5) *	13,210 (16.0)	5,739 (26.3) *
**Fracture type**				
Intracapsular	4,470 (60.4)	1,318 (58.7)	49,988 (59.5)	12,841 (57.1)
Intertrochanteric	2,588 (35.0)	789 (35.1)	29,407 (35.0)	7,986 (35.5)
Subtrochanteric	340 (4.6)	140 (6.2) †	4,653 (5.5)	1,658 (7.4) *
**Prefracture mobility**				
No functional mobility	94 (1.3)	75 (3.4)	852 (1.0)	558 (2.5)
Indoor Only	2,004 (27.4)	881 (39.8)	17,026 (20.5)	7,221 (32.6)
Indoor and Outdoor	5,224 (71.3)	1,258 (56.8) *	65,343 (78.5)	14,382 (64.9) *
**Surgery within the target time**				
Within 36 hours	5,418 (77.7)	1,599 (75.4)	61,117 (77.5)	15,421 (73.2)
Beyond 36 hours	1,556 (22.3)	523 (24.6) †	17,779 (22.5)	5,653 (26.8) *
**Calendar year of admission**				
2014	1,852 (25.0)	610 (27.1)	22,774 (27.1)	6,391 (28.4)
2015	2,774 (37.5)	791 (35.2)	31,499 (37.5)	8,096 (36.0)
2016	2,776 (37.5)	847 (37.7) †	29,814 (35.5)	8,009 (35.6) *
**Weekday of admission**				
Weekday	4,973 (68.1)	1,548 (70.4)	57,175 (69.0)	15,504 (70.5)
Weekend	2,330 (31.9)	650 (29.6) †	25,703 (31.0)	6,494 (29.5) *
**Hospital volume** ^d^				
Low	2,436 (32.9)	809 (36.0)	29,202 (34.7)	8,541 (38.0)
Medium	2,609 (35.2)	749 (33.3)	29,134 (34.6)	7,422 (33.0)
High	2,357 (31.8)	690 (30.7) †	25,751 (30.6)	6,533 (29.0) *

Data are numbers (percentage), otherwise as stated.

^a^ Mobilised early = Mobilised on the day of or day after surgery, Mobilised late = Mobilised 2 days or more after surgery

^b^ Ethnicity–pooled here to avoid reporting small number of patients as a requirement of the data controller, analyses included all variable levels: White, Black or mixed Black, Asian or mixed Asian, Other mixed background

^c^ ASA-grade; 0–1: I–normal healthy individual and II–mild systemic disease that does not limit activity; 2: III–severe systemic disease that limits activity but is not incapacitating; 3–4: IV-incapacitating systemic disease which is constantly life-threatening and V-moribund -not expected to survive 24 hours with or without surgery

^d^ Number of hip fracture surgeries at the treating hospital in the year the patient is treated categorised into the 1^st^, 2^nd^ and 3^rd^ quintiles

* p <0.001; difference between early mobilisation and late mobilisation

† p <0.01; difference between early mobilisation and late mobilisation

**Table 2:** Discharge by timing of mobilisation among patients surgically treated for non-pathological first hip fracture by depression diagnosis.

Mobilisation timing	No of patients n (%)	No of deaths ^a^ n (%)	No. of live discharges ^a^ n (%)	Live discharge rate (95% CI) ^b^
**Patients with a diagnosis of depression**				
Overall	9,659 (100)	345 (3.6)	5,019 (52.0)	36.2 (35.2–37.3)
Mobilised on the day of or day after surgery	7,412 (76.7)	183 (1.9)	4,022 (41.6)	39.5 (38.3–40.8)
Mobilised 2 days or more after surgery	2,247 (23.3)	162 (1.7)	997 (10.3)	27.1 (25.5–28.9)
**Patients without a diagnosis of depression**				
Overall	106,615 (100)	4,621 (4.3)	56,035 (52.6)	39.1 (38.7–39.4)
Mobilised on the day of or day after surgery	84,085 (78.9)	2,548 (2.4)	46,611 (43.7)	43.1 (42.7–43.5)
Mobilised 2 days or more after surgery	22,530 (21.1)	2,073 (1.9)	9,424 (8.8)	26.7 (26.2–27.3)

Proportions calculated from the total number of each subgroup

^a^ At 30 days from surgery

^b^ Per 1000 patient–days.

### Association between mobilisation timing and live discharge

**Fig 1:**
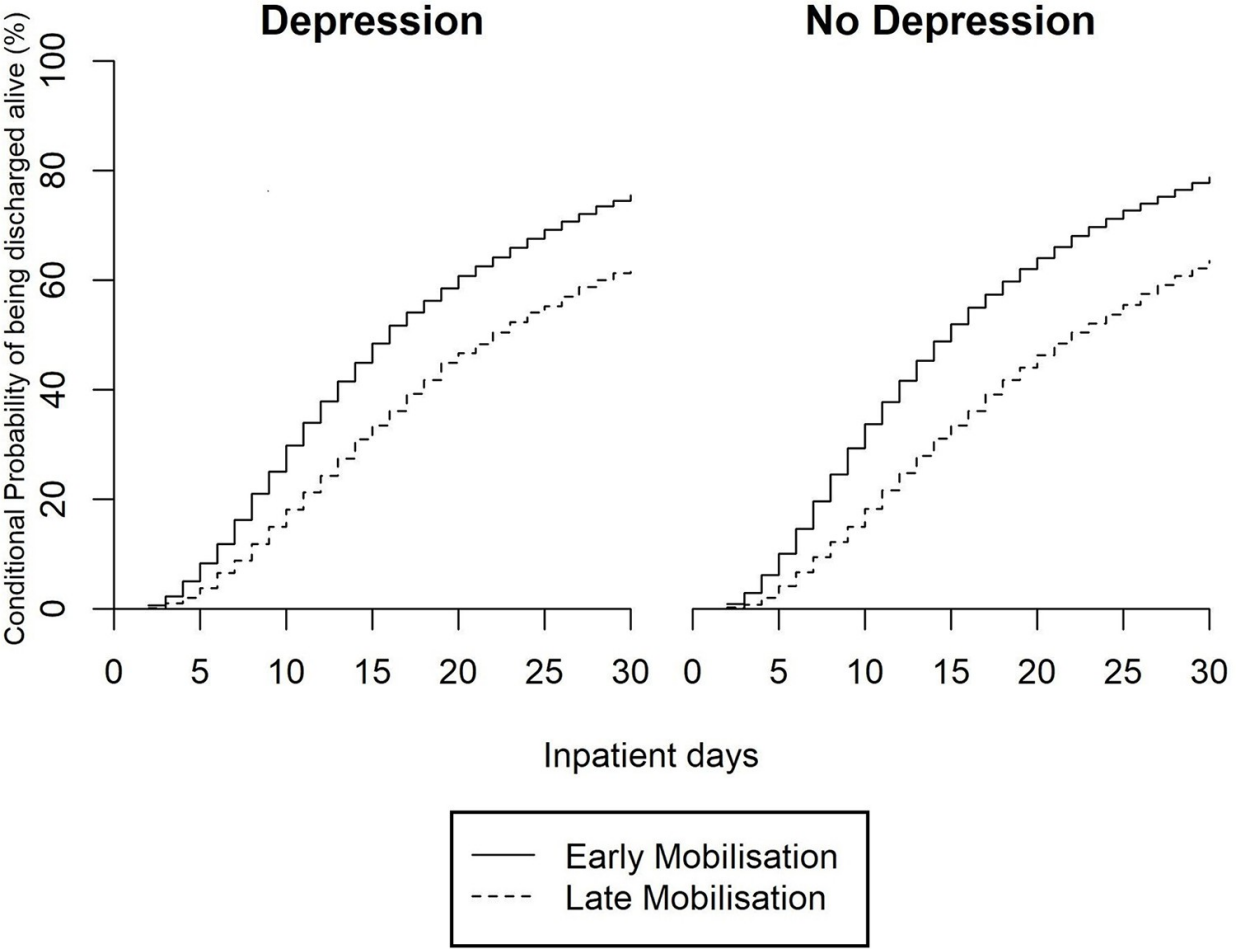
Conditional probability of live discharge by 30-days postoperatively among patients surgically treated for non-pathological first hip fracture by depression diagnosis and timing of mobilisation.

The probabilities of discharge at 30 days after hip fracture surgery, among those with depression, were 0.79 (95% CI: 0.78–0.79) among those mobilised early and 0.64 (95% CI: 0.63–0.64) among those mobilised late conditional on being alive and in hospital. Among those without depression these probabilities were 0.76 (95% CI: 0.74–0.77) and 0.63 (95% CI: 0.60–0.65) among those who mobilised early and mobilised late, respectively (Fig 1, Table 3 (CPF given per 1000 patient-days)).

**Table 3:** Conditional probability of live discharge by timing of mobilisation among all patients surgically treated for non-pathological first hip fracture by depression diagnosis.

Mobilisation timing	30-day CPF, % (95% CI) ^a^	Pepe-Mori test (p value) ^b^	Unadjusted OR of CPF (95% CI)	Adjusted OR of CPF (95% CI) ^c^
**Patients with a diagnosis of depression**				
Overall	72.6 (71.5–73.7)			
Mobilised on the day of or day after surgery	78.7 (78.3–79.1)		1.89 (1.7–2.11)	1.79 (1.56–2.05)
Mobilised 2 days or more after surgery	63.5 (62.7–64.4)	p<0.001	1.00	1.00
**Patients without a diagnosis of depression**				
Overall	75.6 (75.2–75.9)			
Mobilised on the day of or day after surgery	75.5 (74.2–76.9)		2.11 (2.05–2.18)	1.92 (1.84–2.00)
Mobilised 2 days or more after surgery	62.8 (60.2–65.4)	p<0.001	1.0	1.00

Abbreviations: CPF = conditional probability function, CI = confidence interval, OR = odds ratio

^a^ At 30 days from surgery

^b^ Pepe-Mori test p-value. Two–sample test compared the mobilised on the day of or day after surgery group to the mobilised 2 days or more after surgery group

^c^ Adjusted for age, sex, ethnicity, deprivation, ASA grade, prefracture residence, fracture type, mobility prior to hip fracture, hospital surgical volume, timing of surgery, day of admission, calendar year of admission, number of comorbidities.

### Sensitivity analyses

Sensitivity analyses where exposure, subgroup, and outcome data were imputed for patients with these missing data are presented in S2, S3 Tables in S1 File. The unadjusted CPFs by mobilisation timing and depression diagnosis were comparable between the complete case and imputed analyses (S3 Table in S1 File). It was not computationally feasible to calculate the adjusted CPF using the pseudo-values from a jackknife of the CPF in a generalized linear model using multiple imputation analyses for the whole dataset. Therefore, a random subset of 50,000 individuals were analysed using this approach. The adjusted CPF’s from this analysis were comparable between the complete case and imputed analyses (S3 Table in S1 File). Sensitivity analyses where 36,901 patients with no depression code were included in the “No Depression” group produced similar results to the main analysis (S2 Table in S1 File).

## Discussion

In-keeping with previous literature, the results show that early mobilisation after their hip fracture surgery was associated with improved outcomes compared to late mobilisation, and that this association was observed irrespective of depression diagnosis [6, 7, 31, 32]. The probability of discharge by mobilisation timing was similar for those with and without depression.

In this study, 8.31% of the overall population had a diagnosis of depression. This is lower than the often-cited prevalence of depression in hip fracture populations; 9–47% or an average of 23% [10, 33]. This may be explained by the fact that most prevalence studies use self-reported screening tools to measure depression such as the Geriatric Depression Scale (GDS) and the Hospital Anxiety and Depression Scale (HADS). These tools have been shown to overestimate the prevalence of depression [34]. Alternately, the presence of depression (or depressive symptoms) found in the present analysis may be underestimated by the use of formal diagnosis codes. For the systematic review and meta-analysis conducted by Heidari et al. [33], one study in which depression was diagnosed using the ICD-9 coding reported the lowest prevalence of all the 27 included studies: 1.2% [33]. Future research may wish to delineate research which reports on populations of those with a diagnosis of depression using e.g., the ICD-coding/The Diagnostic and Statistical Manual of Mental Disorders (DSM) criteria assessed by a medical professional, and those using self-reported questionnaires related to depressive symptoms.

Mobilisation timing was associated with depression. However, the proportion of patients mobilising early differed by just 2.2% between those with and without depression, which may not be clinically important. This was surprising given the suggestion that depression is associated with a lack of motivation for engaging with physiotherapy [11]. While it has been reported patients with depression have poorer outcomes after orthopaedic surgery than those without depression [35], the results of this study suggest this is not due to differing levels of early mobilisation. It may be that poorer outcomes previously reported relate to ongoing engagement with mobility training beyond its initiation. Alternatively, here, it may be that patients with a diagnosis of depression are more likely to be well supported with medication or non-pharmacological approaches, and their depression less likely to influence their recovery trajectory, than those with subthreshold depressive symptoms.

The relationship between mobilisation timing and discharge by depression status may be confounded by discharge destination. A previous study found live discharge rates were higher after hip fracture surgery among those admitted from nursing/residential care compared to those admitted from home [7]. This is in keeping with earlier research from Sweden whereby patients with hip fracture admitted from nursing homes had shorter length of stays than those admitted from their own homes [36]. Longer length of stays in those admitted from home might be due to several reasons: waiting for a bed or transfer to other care facilities, awaiting care packages or home adaptations [3]. In contrast, shorter stays may be observed for those residing in residential/nursing care given they are returning to a supported environment [37]. In the current study, we adjusted for pre-fracture residence in our analysis however, this would not account for the proportion of patients who change residence from home to nursing/residential care following admission for hip fracture. Moreover, we noted more people admitted from a nursing/residential care home presented with depression than those admitted from home. Further research is required to understand the relationship between prefracture residential status, depression, discharge, and discharge destination after hip fracture surgery.

### Limitations

There were limitations to this study. The hospital records linked to NHFD record an ICD-code for a diagnosis of depression. Therefore, those with subthreshold depressive symptoms, were classified as ‘not depressed’ despite the potential for their symptoms to influence outcomes [38]. There was no information on symptom severity, medication or treatment approaches and responses relating to patient’s depression, therefore, we were unable to investigate if treatment for depression or severity of depression impacted these results. It is likely most patients had preexisting depression however, due to data availability we were unable to assess the proportion of new onset verse preexisting depression in patients with depression. Further, there is the potential for residual confounding by additional variables, including other comorbidities, which may also be associated with early mobilisation. There is potential for bias due to data missingness or data quality [39] however, we noted similar findings for complete and imputed analyses. We did not test the interaction of depression using tests such as the Wald chi-squared or Likelihood Ratio test however, the large number of observations in each group suggests confidence in the interaction results found. The influence of comorbid anxiety on the relationships investigated was not explored and may have impacted the results found given the overlap between anxiety and depression [40]. As the data were collected up until 2016, the results may not be generalisable to post-COVID 19 pandemic health care settings. This data precedes the Best Practice Tariff after which the criterion of patients receiving a physiotherapy assessment on the day of or day after hip fracture was introduced. Therefore, this paper may show more variation in early mobilisation proportions between patients with and without depression or a lower proportion of patients receiving early mobilisation overall. However, from the most recent NHFD Report, 81% of patients were mobilised early [41], this is comparable to the results of this paper. Discharge from hospital was defined as discharge from an acute hospital. Discharge to a rehabilitation hospital or unit was classified as lost to follow up. Therefore, we were unable to determine the impact of depression on the association between early mobilisation and discharge from both an acute and rehabilitation setting. A further limitation was the high proportion of patients who were right censored due to loss to follow-up, and for whom, it was not possible to determine whether their discharge prospects were similar to those not censored. Finally, the results may not be generalisable to those who receive care after hip fracture outside of England or Wales.

## Conclusions

A similar proportion of patients with depression mobilised early after hip fracture surgery when compared to those without a diagnosis of depression. The association between mobilisation timing and time to discharge was observed for patients with and without depression. This was surprising given the reported association between depression and engagement with physiotherapy. Further research is required to understand the relationship between prefracture residential status, depression, discharge, and discharge destination after hip fracture surgery.

## Supporting information

S1 File Supplementary file_PLOS. (DOCX)
