# Supplementary material for: The role of depression in the association between mobilisation timing and live discharge after hip fracture surgery: Secondary analysis of the UK National Hip Fracture Database
Source: PLoS One. 2024 Apr 4;19(4):e0298804. doi: 10.1371/journal.pone.0298804 (PMC10994389; doi:10.1371/journal.pone.0298804)
Supplement: S1 File — (DOCX) [file pone.0298804.s001.docx]

**S1 Table**. Characteristics of 121,503 patients surgically treated for non-pathological hip fracture by depression diagnosis and mobilisation timing and inclusion/exclusion in complete case analysis.

|  | Complete Case | | | | Excluded | | | |
| --- | --- | --- | --- | --- | --- | --- | --- | --- |
|  | Diagnosis of Depression (n=9,659) | | No diagnosis of depression (n=106,615) | | Diagnosis of Depression (n=430) | | No diagnosis of depression (n=4,799) | |
|  | Mobilised early | Mobilised late | Mobilised early | Mobilised late | Mobilised early | Mobilised late | Mobilised early | Mobilised late |
| Age at admission (years) Median (IQR) | 82.0 (74.0-88.0) | 83.0 (76.0-88.0) * | 84.0 (77.0-89.0) | 85.0 (79.0-90.0) * | 81.0 (72.0-86.0) | 81.0 (76.0-87.0) | 83.0 (76.0-88.0) | 85.0 (78.0-89.0) * |
| Number of Comorbidities Median (IQR) | 2.0 (1.0-3.0) | 2.0 (1.0-3.0) * | 1.0 (1.0-2.0) | 2.0 (1.0-3.0) * | 2.0 (1.0-3.0) | 2.0 (1.0-3.0) † | 1.0 (1.0-2.0) | 2.0 (1.0-3.0) * |
| **Sex** | | | | | | | | |
| Women | 5,755 (77.7) | 1,735 (77.2) | 60,959 (72.5) | 15,870 (70.5) | 241 (76.5) | 94 (81.7) | 2,488 (71.5) | 899 (68.1) |
| Men | 1,647 (22.3) | 513 (22.8) | 23,127 (27.5) | 6,625 (29.5) * | 74 (23.5) | 21 (18.3) | 990 (28.5) | 422 (31.9) † |
| **Ethnicity** | | | | | | | | |
| White | 6,258 (99.1) | 1,812 (99.0) | 69,616 (98.6) | 17,743 (98.1) | 245 (97.2) | 93 (100.0) | 2,829 (97.9) | 989 (97.8) |
| Black or mixed Black | 8 (0.1) | 3 (0.2) | 141 (0.2) | 75 (0.4) | 0 (0.0) | 0 (0.0) | 10 (0.3) | 7 (0.7) |
| Asian or mixed Asian | 51 (0.8) | 14 (0.8) | 862 (1.2) | 265 (1.5) | 6 (2.4) | 0 (0.0) | 51 (1.8) | 13 (1.3) |
| Other mixed background | 1 (0.0) | 1 (0.1) | 18 (0.0) | 5 (0.0) * | 1 (0.4) | 0 (0.0) | 1 (0.0) | 2 (0.2) |
| **Deprivation** | | | | | | | | |
| least deprived 10% | 729 (9.9) | 239 (10.7) | 7,095 (8.5) | 1,985 (8.9) | 39 (12.6) | 8 (7.0) | 333 (9.7) | 120 (9.2) |
| less deprived 10-20% | 694 (9.4) | 233 (10.4) | 6,913 (8.3) | 2,018 (9.0) | 34 (11.0) | 7 (6.1) | 300 (8.7) | 138 (10.6) |
| less deprived 20-30% | 737 (10.0) | 241 (10.8) | 7,574 (9.1) | 2,194 (9.8) | 29 (9.4) | 15 (13.0) | 311 (9.1) | 155 (11.9) |
| less deprived 30-40% | 730 (9.9) | 236 (10.6) | 8,225 (9.9) | 2,331 (10.4) | 42 (13.6) | 5 (4.3) | 348 (10.1) | 111 (8.5) |
| less deprived 40-50% | 805 (10.9) | 196 (8.8) | 8,705 (10.4) | 2,404 (10.8) | 29 (9.4) | 14 (12.2) | 340 (9.9) | 130 (10.0) |
| more deprived 40-50% | 753 (10.2) | 255 (11.4) | 9,286 (11.1) | 2,475 (11.1) | 34 (11.0) | 11 (9.6) | 359 (10.5) | 145 (11.2) |
| more deprived 30-40% | 810 (11.0) | 232 (10.4) | 9,110 (10.9) | 2,410 (10.8) | 27 (8.7) | 16 (13.9) | 390 (11.4) | 154 (11.8) |
| more deprived 20-30% | 760 (10.3) | 231 (10.3) | 8,951 (10.7) | 2,248 (10.1) | 25 (8.1) | 13 (11.3) | 334 (9.7) | 141 (10.8) |
| more deprived 10-20% | 701 (9.5) | 193 (8.6) | 8,994 (10.8) | 2,203 (9.9) | 27 (8.7) | 19 (16.5) | 376 (11.0) | 113 (8.7) |
| most deprived 10% | 648 (8.8) | 176 (7.9) † | 8,579 (10.3) | 2,049 (9.2) * | 23 (7.4) | 7 (6.1) † | 338 (9.9) | 93 (7.2) * |
| **ASA Grade ^a^** | | | | | | | | |
| 0-1 | 1,897 (26.2) | 321 (14.7) | 26,908 (32.8) | 4,274 (19.4) | 79 (25.8) | 24 (21.2) | 1,237 (36.5) | 285 (22.3) |
| 2 | 4,378 (60.5) | 1,394 (63.9) | 45,826 (55.9) | 13,130 (59.7) | 191 (62.4) | 63 (55.8) | 1,745 (51.5) | 667 (52.3) |
| 3-4 | 958 (13.2) | 467 (21.4) * | 9,292 (11.3) | 4,594 (20.9) * | 36 (11.8) | 26 (23.0) † | 404 (11.9) | 324 (25.4) * |
| **Prefracture Residence** | | | | | | | | |
| Own home/sheltered housing | 5,353 (74.4) | 1,327 (61.5) | 69,401 (84.0) | 16,086 (73.7) | 232 (80.8) | 66 (65.3) | 2,951 (90.3) | 936 (80.3) |
| nursing care/residential care | 1,838 (25.6) | 829 (38.5) * | 13,210 (16.0) | 5,739 (26.3) * | 55 (19.2) | 35 (34.7) † | 316 (9.7) | 230 (19.7) * |
| **Fracture type** | | | | | | | | |
| Intracapsular | 4,470 (60.4) | 1,318 (58.7) | 49,988 (59.5) | 12,841 (57.1) | 186 (59.0) | 78 (67.8) | 2,058 (59.2) | 765 (58.0) |
| Intertrochanteric | 2,588 (35.0) | 789 (35.1) | 29,407 (35.0) | 7,986 (35.5) | 117 (37.1) | 32 (27.8) | 1,224 (35.2) | 470 (35.6) |
| Subtrochanteric | 340 (4.6) | 140 (6.2) † | 4,653 (5.5) | 1,658 (7.4) * | 12 (3.8) | 5 (4.3) | 194 (5.6) | 84 (6.4) |
| **Prefracture mobility** | | | | | | | | |
| No functional mobility | 94 (1.3) | 75 (3.4) | 852 (1.0) | 558 (2.5) | 4 (1.3) | 5 (4.5) | 24 (0.7) | 35 (2.7) |
| Indoor Only | 2,004 (27.4) | 881 (39.8) | 17,026 (20.5) | 7,221 (32.6) | 84 (27.3) | 43 (38.4) | 668 (19.5) | 385 (30.2) |
| Indoor and Outdoor | 5,224 (71.3) | 1,258 (56.8) * | 65,343 (78.5) | 14,382 (64.9) * | 220 (71.4) | 64 (57.1) † | 2,736 (79.8) | 856 (67.1) * |
| **Surgery within the target time** | | | | | | | | |
| Within 36 hours | 5,418 (77.7) | 1,599 (75.4) | 61,117 (77.5) | 15,421 (73.2) | 214 (73.8) | 64 (63.4) | 2,266 (72.8) | 806 (67.4) |
| Beyond 36 hours | 1,556 (22.3) | 523 (24.6) † | 17,779 (22.5) | 5,653 (26.8) * | 76 (26.2) | 37 (36.6) † | 847 (27.2) | 390 (32.6) * |
| **Calendar year of admission** | | | | | | | | |
| 2014 | 1,852 (25.0) | 610 (27.1) | 22,774 (27.1) | 6,391 (28.4) | 76 (24.1) | 48 (41.7) | 1,154 (33.2) | 464 (35.1) |
| 2015 | 2,774 (37.5) | 791 (35.2) | 31,499 (37.5) | 8,096 (36.0) | 163 (51.7) | 44 (38.3) | 1,481 (42.6) | 571 (43.2) |
| 2016 | 2,776 (37.5) | 847 (37.7) † | 29,814 (35.5) | 8,009 (35.6) * | 76 (24.1) | 23 (20.0) † | 843 (24.2) | 286 (21.7) |
| **Weekday of admission** | | | | | | | | |
| Weekday | 4,973 (68.1) | 1,548 (70.4) | 57,175 (69.0) | 15,504 (70.5) | 143 (66.5) | 35 (70.0) | 1,389 (67.6) | 392 (66.4) |
| Weekend | 2,330 (31.9) | 650 (29.6) † | 25,703 (31.0) | 6,494 (29.5) * | 72 (33.5) | 15 (30.0) | 667 (32.4) | 198 (33.6) |
| **Hospital volume ^b^** | | | | | | | | |
| Low | 2,436 (32.9) | 809 (36.0) | 29,202 (34.7) | 8,541 (38.0) | 107 (34.0) | 58 (50.4) | 1,357 (39.0) | 639 (48.4) |
| Medium | 2,609 (35.2) | 749 (33.3) | 29,134 (34.6) | 7,422 (33.0) | 116 (36.8) | 38 (33.0) | 1,169 (33.6) | 401 (30.4) |
| High | 2,357 (31.8) | 690 (30.7) † | 25,751 (30.6) | 6,533 (29.0) * | 92 (29.2) | 19 (16.5) † | 952 (27.4) | 281 (21.3) * |

Data are numbers (percentage), otherwise as stated.

a ASA-grade; 0-1: I – normal healthy individual and II – mild systemic disease that does not limit activity; 2: III – severe systemic disease that limits activity but is not incapacitating; 3-4: IV-incapacitating systemic disease which is constantly life-threatening and V-moribund -not expected to survive 24 hours with or without surgery

b Number of hip fracture surgeries at the treating hospital in the year the patient is treated categorised into the 1st, 2nd and 3rd quintiles

* p <0.001; difference between early mobilisation and late mobilisation

† p <0.01; difference between early mobilisation and late mobilisation

**S2 Table:** Conditional probability analysis where n=36,901 with no depression code are included in the “No Depression” Group

| Mobilisation timing | 30-day CPF,  % (95% CI) | Pepe-Mori test (p value)§ | Unadjusted OR  of CPF (95% CI) | Adjusted OR  of CPF (95% CI)ǁ |
| --- | --- | --- | --- | --- |
|  | Patients with a diagnosis of depression | | | |
| Overall | 725 (713-737) |  |  |  |
| Mobilised on the day of or day after surgery | 755 (742-769) |  | 2.44 (1.87-3.18) | 1.61 (1.1-2.36) |
| Mobilised 2 days or more after surgery | 628 (602-654) | p<0.001 | 1.00 | 1.00 |
|  | Patients without a diagnosis of depression | | | |
| Overall | 757 (754-760) |  |  |  |
| Mobilised on the day of or day after surgery | 789 (785-792) |  | 2.21 (2.05-2.38) | 1.90 (1.68-2.15) |
| Mobilised 2 days or more after surgery | 639 (631-646) | p<0.001 | 1.00 | 1.00 |

Abbreviations: CPF = conditional probability function, CI = confidence interval, OR = odds ratio

**S3 Table.** Conditional Probability Analysis Imputed Datasets (n=50,000)

| Mobilisation timing | 30-day CPF,  % (95% CI) | Pepe-Mori test (p value) | Unadjusted OR  of CPF (95% CI) | Adjusted OR  of CPF (95% CI) |
| --- | --- | --- | --- | --- |
|  | Patients with a diagnosis of depression | | | |
| Overall | 727 (716-739) |  |  |  |
| Mobilised on the day of or day after surgery | 756 (744-768) |  | 1.84 (1.54-2.18) | 1.71 (1.42-2.05) |
| Mobilised 2 days or more after surgery | 638 (612-665) | p<0.001 | 1.00 | 1.00 |
|  | Patients without a diagnosis of depression | | | |
| Overall | 757 (754-761) |  |  |  |
| Mobilised on the day of or day after surgery | 787 (784 -791) |  | 2.11 (2.00-2.22) | 1.97 (1.86-2.08) |
| Mobilised 2 days or more after surgery | 648 (640-656) | p<0.001 | 1.00 | 1.00 |

Abbreviations: CPF = conditional probability function, CI = confidence interval, OR = odds ratio
